# Effects of Replacing Fishmeal with Soybean Meal on Intestinal Histology, Antioxidation, Endoplasmic Reticulum Stress, Inflammation, Tight Junction, and Microbiota in Olive Flounder (*Paralichthys olivaceus*)

**DOI:** 10.3390/ani15192895

**Published:** 2025-10-03

**Authors:** Zhenxia Su, Yanjie Zhang, Chaoqing Wei, Fengxiang Zhang, Lei Wang, Yaxuan Li, Zhengqiu Zhang, Jianhe Xu, Zhiguo Dong, Hua Mu

**Affiliations:** 1 Jiangsu Key Laboratory of Marine Bioresources and Environment, Jiangsu Key Laboratory of Marine Biotechnology, Jiangsu Ocean University, Lianyungang 222005, China; szx08042023@163.com (Z.S.); 2023120120@jou.edu.cn (Y.Z.); zfx15719988298@163.com (F.Z.); 15750346500@163.com (Y.L.); 19732893195@163.com (Z.Z.); jianhexu218@163.com (J.X.); dzg7712@163.com (Z.D.); 2 Co-Innovation Center of Jiangsu Marine Bio-Industry Technology, Jiangsu Ocean University, Lianyungang 222005, China; 3 Marine Resource Development Institute of Jiangsu, Lianyungang 222005, China; 4 School of Ocean, Yantai University, Yantai 264005, China; sanshitongxue@126.com

**Keywords:** fishmeal, soybean meal, endoplasmic reticulum stress, inflammation, tight junction, intestinal microbiota, olive flounder

## Abstract

**Simple Summary:**

Fishmeal is a common ingredient in fish diets, but it is currently facing global shortages and price increases. Meanwhile, widespread application of fishmeal in aquafeeds could lead to severe environmental, ecological, and ethical challenges. It is essential to explore alternative protein sources for fishmeal. Soybean meal presents a promising alternative protein source, though high inclusion levels may negatively impact fish, particularly marine carnivorous species. This research evaluated the effectiveness of soybean meal for olive flounder *Paralichthys olivaceus* by examining its effects on intestinal histology, antioxidation, endoplasmic reticulum stress (ERS), inflammation, tight junction, and microbial community composition. The results showed that when the proportion of soybean meal replacing fishmeal reaches 24% or higher, it impaired intestinal health in olive flounder by reducing intestinal antioxidant capacity, inducing intestinal ERS, inflammatory response, barrier disruption, and microbial dysbiosis. These results could provide valuable insights for optimizing feed formulations in olive flounder aquaculture.

**Abstract:**

A limited supply and price shortages of fishmeal with the expansion of aquaculture make it necessary to seek alternative protein sources. Soybean meal (SM) has been the widely preferred replacer for fishmeal in fish diets. Nevertheless, this substitution, especially when given at high doses, potentially shows adverse impact on fish intestinal health. This study aimed to investigate the effect of replacing fishmeal with SM on intestinal health in olive flounder (*Paralichthys olivaceus*). A 56-day feeding trial was conducted with 450 juvenile fish (initial weight: 6.32 ± 0.01 g) randomly allocated to five diets with graded SM replacement: 0% (FM), 12% (SM12), 24% (SM24), 36% (SM36), and 48% (SM48). The results demonstrated that concentrations of glucose, total triglyceride, and low-density lipoprotein cholesterol increased, whereas total protein and high-density lipoprotein cholesterol contents, and lysozyme activity decreased in serum with increasing dietary SM levels. Meanwhile, total antioxidant capacity and superoxide dismutase activity significantly decreased at replacement levels exceeding 24%, accompanied by elevated malondialdehyde concentration (*p* < 0.05). Compared with the FM group, the SM24, SM36, and SM48 groups showed significantly reduced VH and increased lamina propria width (*p* < 0.05). Increasing dietary SM levels upregulated expression of genes related to endoplasmic reticulum stress (ERS) (*chop*, *perk*, and *grp78*), inflammation (*tnf-α* and *il-6*), and apoptosis (*bax*, *casp3*, *casp6*, and *casp9*), while downregulated anti-inflammatory cytokines (*il-10* and *tgf-β1*) and tight junction-related genes (*zo-1*, *zo-2*, *claudin-5*, *ocln*, *muc-13*, and *muc-15*) in the intestine (*p* < 0.05). There were significant differences in the abundances of intestinal microbiota at both the phylum and genus levels among the FM, SM24, and SM36 groups (*p* < 0.05), but the clusters and microbiota composition of the SM24 group were more similar to those of the FM group. In conclusion, replacing 24% of fishmeal with SM induced intestinal dysfunction through evoking ERS, inflammation, barrier disruption, and microbial dysbiosis in olive flounder.

## 1. Introduction

Aquaculture has emerged as the fastest growing sector of food production and is projected to sustain its growth trajectory in the future [1,2]. Fishmeal is traditionally served as the main protein source in aquafeeds. However, widespread application of fishmeal in fish diets could pose severe environmental, ecological, and ethical challenges [3,4]. Meanwhile, the limited supply of fishmeal is unable to meet the growing demand from the feed industry and aquaculture. Consequently, research on suitable alternatives to fishmeal has become an international priority. Plant-based protein sources are widely preferred due to their cost-effectiveness, ready availability, and stable supply characteristics. Among various plant protein sources, soybean meal (SM) is particularly favored by industries and researchers, owing to its relatively balanced amino acid profile and other nutritional advantages [5]. However, SM contains various antinutritional factors, including saponins, phytic acid, and lectins, which may cause adverse impacts on fish, especially marine carnivorous fish species [6,7].

Previous studies have consistently demonstrated that excessive inclusion of SM in fish diets elicits adverse effects on intestinal health and immune function [8,9,10]. High SM levels disrupt the integrity of the intestinal structure, leading to histological alterations such as tissue disruption, villus atrophy, goblet cell depletion, and expansion of the lamina propria [11]. These structural changes subsequently compromise intestinal barrier function, evidenced by increased intestinal permeability and impaired mucosal defense, which in turn triggers intestinal inflammation [12,13].

It is known that the integrity of intestinal structure is crucial for the intestinal health of fish. The integrity of intestinal structure can be compromised by cellular damage (through mechanisms such as oxidative damage and apoptosis) and intracellular structure (composed of tight junction proteins) [14]. Diets containing a high level of SM have been found to reduce superoxide dismutase (SOD) activity and elevate malondialdehyde (MDA) content in aquatic animals [15]. Concurrently, SM-induced oxidative stress activates the endoplasmic reticulum stress (ERS), disrupting protein folding and cellular homeostasis [16], whereas, sustained ERS triggers inflammatory response and apoptosis via the unfolded protein response (UPR), with reactive oxygen species generated by inflammation exacerbating endoplasmic reticulum dysfunction to form a self-perpetuating vicious cycle [17]. A study on turbot (*Scophthalmus maximus* L.) indicated that a high level of dietary SM upregulated apoptosis-related gene expression while downregulated the expression levels of tight junction-related genes, directly contributing to intestinal epithelial damage [18].

The intestinal microbiota plays a crucial role in maintaining host health by participating in nutrient metabolism, intestinal barrier, and inflammation [19,20,21]. However, dysbiosis of the intestinal microbiota can lead to immune response imbalance and disruption of the intestinal barrier, resulting in diseases. It has been concluded that high SM diets alter the composition of gut microbiota in large yellow croaker (*Larimichthys crocea*), leading to an increase in potentially pathogenic bacteria and a decrease in beneficial microbes [22]. The stability of gut microbiota is largely influenced by the interaction between the microbiota and gut environment, including change in dietary protein sources [23]. Therefore, understanding how SM impacts fish metabolic functions and microbiota–host interactions is essential for optimizing aquafeed formulations.

Olive flounder (*Paralichthys olivaceus*) is a carnivorous species and has experienced remarkable growth in its aquaculture scale across northern China, mainly due to its delicious taste and rapid growth ability. Our previous studies found that replacing 36% of fishmeal with SM significantly reduced the growth performance and muscle quality [24,25]. However, no studies have comprehensively evaluated the effect of high SM supplementation on the intestinal health of olive flounder, specifically integrating assessments of gut histology, antioxidation, ERS, inflammation, tight junction integrity, and intestinal microbiota composition. The purpose of this study was to investigate the effect of replacing fishmeal with different SM levels on the intestinal health of olive flounder. By elucidating how SM induces intestinal damage, this study aims to optimize the application of SM in aquatic feed.

## 2. Materials and Methods

### 2.1. Ethics Statement

All experimental protocols and procedures for animal husbandry and handling conducted in this study were reviewed and approved by the Animal Care Committee of Jiangsu Ocean University (protocol no. 2020-37; approval date: 1 September 2019).

### 2.2. Experimental Diets and Design

Five isonitrogenous (50% crude protein) and isolipidic (10% crude lipid) experimental diets were formulated in this study (Table 1). Fishmeal, SM, and wheat gluten were used as the major protein sources. Fish oil and soybean lecithin were used as the major lipid sources. The control diet (named FM group) contained 60% fishmeal. Based on the control diet, 12%, 24%, 36%, and 48% of fishmeal were replaced by SM to design the other four diets referred to as the SM12, SM24, SM36, and SM48, respectively. The ingredients were ground to pass an 80-mesh sieve, mixed thoroughly by gradient dilution method, and blended with fish oil and water. Pellets (2.0 mm in diameter) were made automatically by a pellet-making machine and dried in a ventilated oven at 40 °C. The pellets were stored at −20 °C before use.

### 2.3. Feeding Trial and Sample Collection

Healthy olive flounder juveniles were obtained from Rizhao Jinhang Fishery Co., Ltd. (Rizhao, China), and were acclimated for two weeks before being randomly distributed into 15 tanks (30 fish per tank, three replicates per diet, initial weight: 6.32 ± 0.01 g). Feeding was performed twice a day (06:00 and 18:00) for 56 days. Throughout the trial, water quality parameters were maintained as follows: water temperature 19.0 ± 0.5 °C, pH 7.9 ± 0.2, dissolved oxygen 6.7 ± 0.3 mg/L, salinity 26.5 ± 0.8, and ammonia nitrogen < 0.1 mg/L.

Once the feeding experiment concluded, all fish in each tank were weighed and tallied to calculate growth-related indicators. Fish were anesthetized with eugenol (1:10,000 dilution; 99% purity, Shanghai Reagent, Shanghai, China). Blood samples were harvested from the caudal vein of six fish per tank, followed by centrifugation at 4000 rpm under 4 °C for 10 min. The resultant serum was immediately preserved at −80 °C for subsequent biochemical assays. The mid-intestine with potential susceptibility to feed nutrients is an intermediate region for both absorption and immune-related processes. The mid-intestines of these six fish from each tank were thus separated as target tissue, with three samples fixed in 4% paraformaldehyde solution for histological evaluation, while the other three were immediately fixed in liquid nitrogen and stored at −80 °C for antioxidant enzyme activity analysis. Additionally, the mid-intestine samples of another three fish per tank were collected for gene expression examination. For intestinal microbiota analysis, the intestinal contents of eighteen fish from each group (FM, SM24, and SM36 groups) were aseptically collected into sterile tubes, flash frozen in liquid nitrogen and kept at −80 °C.

### 2.4. Serum Biochemical Parameters and Antioxidant Enzyme Activities Analyses

According to the instructions of commercial kits (Nanjing Jiancheng Bioengineering Institute, Nanjing, China), serum biochemical parameters were determined, including the contents of total protein (TP), albumin (ALB), glucose (GLU), total triglyceride (TG), total cholesterol (TC), high-density lipoprotein cholesterol (HDL-C), and low-density lipoprotein cholesterol (LDL-C), as well as lysozyme (LZM) activity.

The catalase (CAT) and superoxide dismutase (SOD) activities, and contents of total antioxidant capacity (T-AOC) and malondialdehyde (MDA) in mid-intestine were detected using commercially available kits (Nanjing Jiancheng Bioengineering Institute, Nanjing, China). The determination steps were carried out in strict accordance with the kit instructions.

### 2.5. Intestinal Histological Analysis

The fixed mid-intestine specimens were dehydrated with gradient ethanol and embedded in paraffin wax. The 5-micron-thick paraffin sections were cut, mounted onto glass slides, and then stained with hematoxylin and eosin (H&E) and periodic acid-Schiff (PAS). The villus height (VH), enterocyte height (EH), and lamina propria width (LPW) were observed under a light microscope (Olympus, DP72) for image acquisition. The VH, EH, and LPW of intestinal villi in different groups were measured by ImageJ 1.47v.

### 2.6. Quantitative Real-Time Polymerase Chain Reaction (qRT-PCR)

Total RNA was extracted using a total RNA isolation kit (Sangon Biotech (Shanghai) Co., Ltd., Shanghai, China). The integrity of RNA was measured by electrophoresis on a 1.2% agarose gel, and the concentration was quantified using GeneQuant pro (GE Pharmacia, Arlington Heights, IL, USA). Then, the extracted RNA was reverse-transcribed to cDNA with the reverse transcription kit (Vazyme Biotech Co., Ltd., Nanjing, China). The qRT-PCR was performed with a thermal cycle procedure as follows: denaturation at 94 °C for 30 s, followed by 40 cycles of 94 °C for 10 s, and 60 °C for 30 s. The relative quantitative method (2^−ΔΔCT^) [26] was used to calculate the gene expression levels of target genes, with *β-actin* as reference gene. The primer sequences of target genes related to ERS, inflammatory response, apoptosis, and tight junction are presented in Table 2.

**Table 2:** Primer sequences of target genes in real-time PCR analysis.

Target Genes	Primer Sequence Forward (5′-3′)	Primer Sequence Reverse (5′-3′)	Source
Endoplasmic reticulum stress			
*chop*	CGGCCAAAAAGAGTCGCAAA	TCTCCGCTTTCAATCGCTCA	XM020096956
*perk*	CTACCACCTACATCGTCCGC	ACCGGCTCAAAGTCAGTCAG	XM020105998
*grp78*	GTCGTGAGGTTGAGAAGGCA	TCATGGTGGAACGGAACAGG	DQ662232
Inflammatory response			
*il-10*	TTTCAAAAGCCCGTTTGCGT	TTGGTTTCCTCCGTCACTCC	KF025663
*tgf-β1*	CAGCGAACACGAGCCAAACAC	TGTTCTGAGGGATGGACATGGTG	[27]
*tnf-α*	AGGGTATGGCTCTTCACGG	AAAGGCCCCCCAGCACCTTACACAT	AB040449
*il-6*	AATACGAGCCCACCGACAG	TGACCAGGGTTCCTCATCTTT	DQ884914
Apoptosis			
*bax*	GAGACACGGAGACAGCAAT	TTAGTGGGACTGAGTGAGGA	XM_020094597
*casp3*	CTGACTTCCTCTACGCCTTCT	AAACTCTACCGCCACCTTG	JQ394697
*casp6*	AACCTAACGGAGACAGATGC	CTGTGATGTCCTGAATAGCG	XM_020101241
*casp9*	CAAGCCTTTCCATTATTCCT	CAGTGGGTGTAGCAGGTTGTA	XM_020089046
Tight junction			
*zo-1*	GGCACCAGGGTTTGGCTTCG	CGTCCGCTCCGTGTCTCAT	XM_020078978
*zo-2*	CGCAGTGGTATCCCATCGT	AGTGTAAGTCCCGCCCTCAT	XM_020105133
*claudin-5*	GTCTGTTTGTGCTGGTGCCTCT	CTCTTGGTCGGTGCGTATGTT	XM _020099317
*claudin-15*	TTCCCTTCACTACTCGCTTTAA	CATACCAGGACACTGAGACCAT	XM_020095475
*ocln*	TCTTTGCTCTGAAGACCCGC	ATTGTTCACCCATGCCTCCA	[27]
*muc-13*	ATGCGTCGCTTGTCCCACT	CATAAGGTTTCCCGATGTCT	XM_020106590
*muc-15*	GTCCAGTAGCGAAGTGAGTGA	GTTGTTGCGTAGCGTTGAT	XM_020099418
Reference gene			
*β-actin*	GGAAATCGTGCGTGACATTAAG	CCTCTGGACAACGGAACCTCT	HQ386788

Abbreviations: *chop*, C/EBP homologous protein; *perk*, protein kinase R (PKR)-like endoplasmic reticulum kinase; *grp78*, glucose-regulated protein 78; *il-10*, interleukin-10; *tgf-β1*, transforming growth factor-β1; *tnf-α*, tumor necrosis factor-α; *il-6*, interleukin-6; *bax*, bcl-2-associated X protein; *casp 3*, cysteinyl aspartate specific proteinase 3; *casp 6*, cysteinyl aspartate specific proteinase 6; *casp 9*, cysteinyl aspartate specific proteinase 9; *zo-1*, zonula occludens-1; *zo-2*, zonula occludens-2; *ocln*, occludin; *muc-13*, mucin-13; *muc-15*, mucin-15.

### 2.7. Intestinal Microbiota Analysis

The total genomic DNA was extracted from intestinal contents using the PowerSoil DNA Isolation Kit (MoBio, Carlsbad, CA, USA). The concentration of DNA was detected by GeneQuant pro (GE Pharmacia, Arlington Heights, IL, USA), and DNA quality was measured by electrophoresis on a 1.2% agarose gel. The target strips were recovered using the Qiagen Gel Extraction Kit (Qiagen, Hilden, Germany). MiSeq sequencing (Illumina, San Diego, CA, USA) and library construction were performed at Gene Denovo Biotechnology Co., Ltd., Guangzhou, China. The bacteria genomic DNA was amplified with the 515F and 806R primers specific to the V4 hypervariable regions of the 16S rDNA gene with the barcode. PCR was performed on the hypervariable regions V3~V4 of the 16S rRNA to taxonomically identify the bacteria. Raw reads were filtered for quality, spliced, and subjected to chimera removal following the methods of Callahan et al. [28] to obtain the optimized sequences. Uparse software (v7.0.1001) was utilized to cluster optimized sequences with ≥97% similarity into the same OTUs. The representative sequences of each OTU were screened, and annotation for taxonomic information was annotated based on the Silva Database (Version 132) using the Mothur algorithm (v1.43.0) [29]. To analyze the construction of phylogenetic relationships, the abundance information of OTUs was normalized using a standard based on the sequence number of the sample with the least number of sequences, with the MUSCLE software (v3.8.31) [30]. Principal coordinates analysis (PCoA), Non-Metric Multi-Dimensional Scaling (NMDS) analysis, and Unweighted Pair-group Method with Arithmetic Mean (UPGMA) analysis based on weighted UniFrac distances were included to compare the similarity of community composition among samples. The R software (v2.15.3) was utilized to visualize NMDS using the vegan package (v2.5-7) and PCoA using the WGCNA package (v1.69), stats packages (v2.15.3), and ggplot2 packages (v3.3.6). Additionally, NMDS analysis, PCoA, and UPGMA based on weighted UniFrac (R software, v2.15.3) distances were included to compare the community composition similarity of samples.

### 2.8. Statistical Analysis

All data were subjected to one-way analysis of variance (ANOVA) using SPSS 22.0 (SPSS Inc., Chicago, IL, USA). Differences among the means were assessed using Tukey’s multiple range tests. The *p* < 0.05 was regarded as statistically significant. The results were expressed as means ± SE (standard error).

## 3. Results

### 3.1. Growth and Feed Performance

As reported previously [24], the fish fed SM36 and SM48 exhibited significantly reduced final body weight, weight gain rate, specific growth rate, feed efficiency ratio, and protein efficiency ratio than those of the FM group (*p* < 0.05), whereas replacing 12% and 24% fishmeal with SM did not significantly affect the aforesaid parameters (*p* > 0.05). Meanwhile, dietary SM showed no significant difference on the survival rate of fish (*p* > 0.05).

### 3.2. Serum Biochemical Parameters

Serum biochemical parameters of olive flounder fed with experimental diets are presented in Table 3. The serum TP content in all SM-supplemented groups was significantly lower than that in the FM group (*p* < 0.05). The GLU content of fish fed with SM36 and SM48 was significantly higher than that of the other groups (*p* < 0.05). Compared with the FM group, the TG content significantly increased when substitution of dietary fishmeal with SM surpassed 12% (*p* < 0.05). When the substitution level with SM was above 12%, the HDL-C content and LZM activity were substantially lower than those in the FM group (*p* < 0.05). The LDL-C content was markedly higher than that in the FM group when the substitution of dietary fishmeal with SM surpassed 36% (*p* < 0.05). There were no significant differences in the serum ALB and TC contents (*p* > 0.05).

### 3.3. Intestinal Morphology

**Figure 1:**
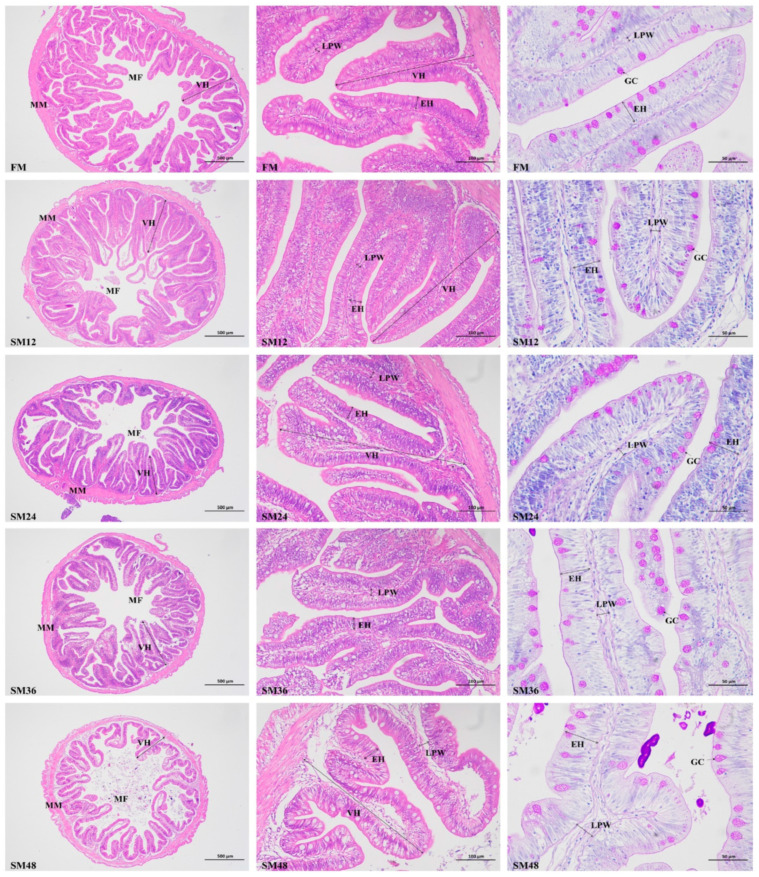
Intestinal morphology of olive flounder fed with the experimental diets (40 ×, scale bar = 500 μm; 200 ×, scale bar = 100 μm; 400 ×, scale bar = 50 μm). MM, muscularis mucosa; MF, mucosal fold; VH, villus height; EH, enterocyte height; LPW, lamina propria width; GC, goblet cell.

**Figure 2:**
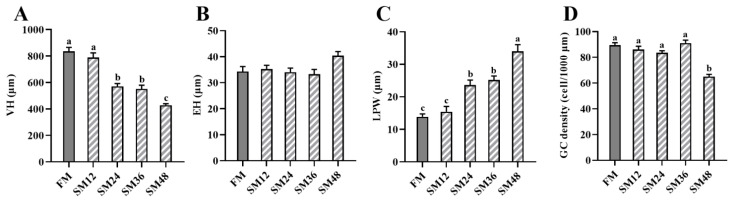
Intestinal morphological parameters of olive flounder fed with the experimental diets. (**A**) VH, villus height; (**B**) EH, enterocyte height; (**C**) LPW, lamina propria width; (**D**) GC, goblet cell. Values are presented as the means ± standard error (*n* = 3). Different lowercases above the bars indicate significant differences (*p* < 0.05). The gray columns represent the FM group (the control diet containing 60% fishmeal), and the striped columns represent the SM groups. These SM groups include SM12, SM24, SM36, and SM48, where 12%, 24%, 36%, and 48% of fishmeal in the control diet was replaced by soybean meal, respectively.

The histological sections of the mid-intestines are illustrated in Figure 1, and the measurements of intestinal morphology are shown in Figure 2. Compared with the FM group, VH was significantly decreased when the substitution of dietary fishmeal with SM exceeded 12% (*p* < 0.05). The LPW of fish fed with SM24, SM36, and SM48 was significantly higher than that of the other groups (*p* < 0.05). The GC density in the SM48 group was significantly lower than that in the other groups (*p* < 0.05).

### 3.4. Intestinal Antioxidant Enzymes Activities

As presented in Table 4, in comparison to the FM group, the T-AOC in intestine of the SM24, SM36, and SM48 groups exhibited a significant decrease (*p* < 0.05). Fish fed diets with SM36 and SM48 had significantly lower SOD activity in intestine than that in other groups (*p* < 0.05). The MDA content in intestine significantly increased when the substitution of dietary fishmeal with SM surpassed 24% (*p* < 0.05). However, no significant difference in CAT activity in intestine was observed among all groups (*p* > 0.05).

### 3.5. The Expression of Genes Related to ERS and Inflammation in Intestine

**Figure 3:**
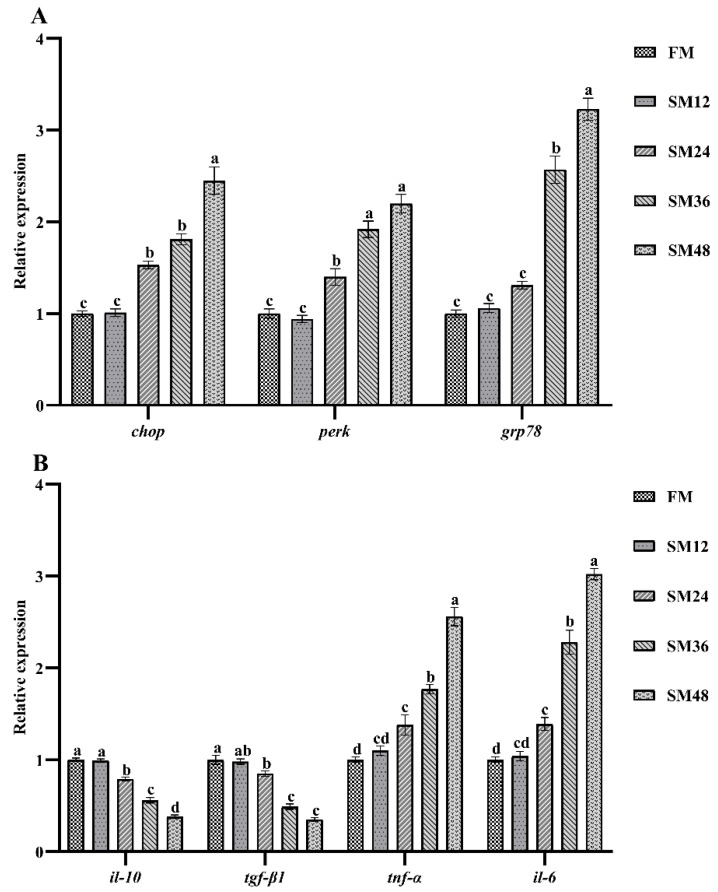
The relative expression levels of genes related to endoplasmic reticulum stress (**A**) and inflammation (**B**) in intestine of olive flounder fed with the experimental diets. Values are presented as the means ± standard error (*n* = 3). Different lowercases above the bars indicate significant differences (*p* < 0.05). *chop*, C/EBP homologous protein; *perk*, protein kinase R (PKR)-like endoplasmic reticulum kinase; *grp78*, glucose-regulated protein 78; *il-10*, interleukin-10; *tgf-β1*, transforming growth factor-β1; *tnf-α*, tumor necrosis factor-α; *il-6*, interleukin-6.

The mRNA levels of genes related to ERS and inflammation in intestine are shown in Figure 3. The expression levels of C/EBP homologous protein (*chop*) and protein kinase R (PKR)-like endoplasmic reticulum kinase (*perk*) were significantly increased when the substitution of dietary fishmeal with SM exceeded 12% (*p* < 0.05). The expression level of glucose-regulated protein 78 (*grp*78) was significantly higher in the SM36 and SM48 groups than in the other groups (*p* < 0.05) (Figure 3A). Compared with the FM group, the gene expression levels of interleukin-10 (*il-10*) and transforming growth factor-β1 (*tgf-β1*) were significantly decreased when the substitution of dietary fishmeal with SM exceeded 12%, whereas the mRNA levels of tumor necrosis factor-α (*tnf-α*) and interleukin-6 (*il-6*) were significantly increased in the SM24, SM36, and SM48 groups (*p* < 0.05) (Figure 3B).

### 3.6. The Expression of Genes Related to Apoptosis and Tight Junction in Intestine

**Figure 4:**
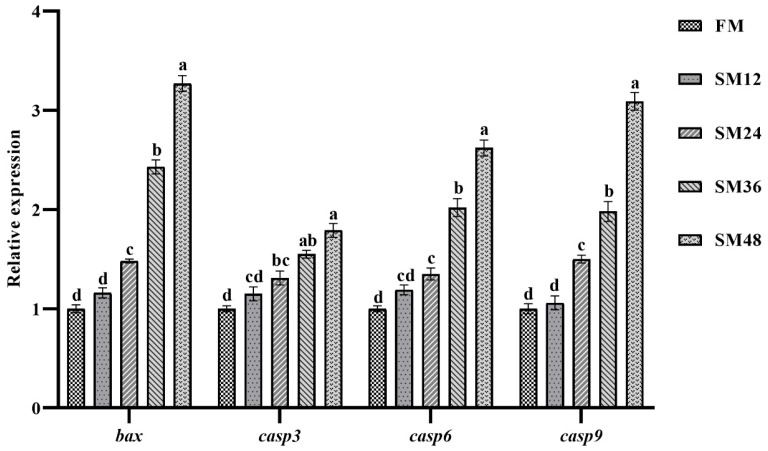
The relative expression levels of apoptosis-related genes in intestine of olive flounder fed with the experimental diets. Values are presented as the means ± standard error (*n* = 3). Different lowercases above the bars indicate significant differences (*p* < 0.05). *bax***,** bcl-2-associated X protein; *casp 3*, cysteinyl aspartate specific proteinase 3; *casp 6*, cysteinyl aspartate specific proteinase 6; *casp 9*, cysteinyl aspartate specific proteinase 9.

**Figure 5:**
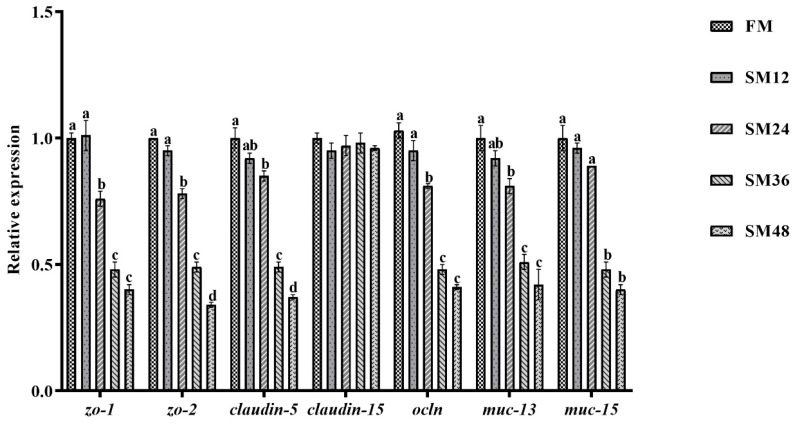
The relative expression levels of tight junction-related genes in intestine of olive flounder fed with the experimental diets. Values are presented as the means ± standard error (*n* = 3). Different lowercases above the bars indicate significant differences (*p* < 0.05). *zo-1*, zonula occludens-1; *zo-2*, zonula occludens-2; *ocln*, occludin; *muc-13*, mucin-13; *muc-15*, mucin-15.

As shown in Figure 4, the relative expression levels of bcl-2-associated X protein (*bax*), cysteinyl aspartate specific proteinase 3 (*casp3*), cysteinyl aspartate specific proteinase 6 (*casp6*), and cysteinyl aspartate specific proteinase 9 (*casp9*) in intestine were significantly increased when the substitution of dietary fishmeal with SM surpassed 12% (*p* < 0.05). As illustrated in Figure 5, compared with the FM group, the expression levels of zonula occludens-1 (*zo-1*), zonula occludens-2 (*zo-2*), *claudin-5*, occluding (*ocln*), and mucin-13 (*muc-13*) in intestine were notably reduced when substitution of dietary fishmeal with SM exceeded 12% (*p* < 0.05). The mucin-15 (*muc-15*) expression level in the SM36 and SM48 groups was significantly lower than that in the other groups (*p* < 0.05).

### 3.7. Intestinal Microbiota

**Figure 6:**
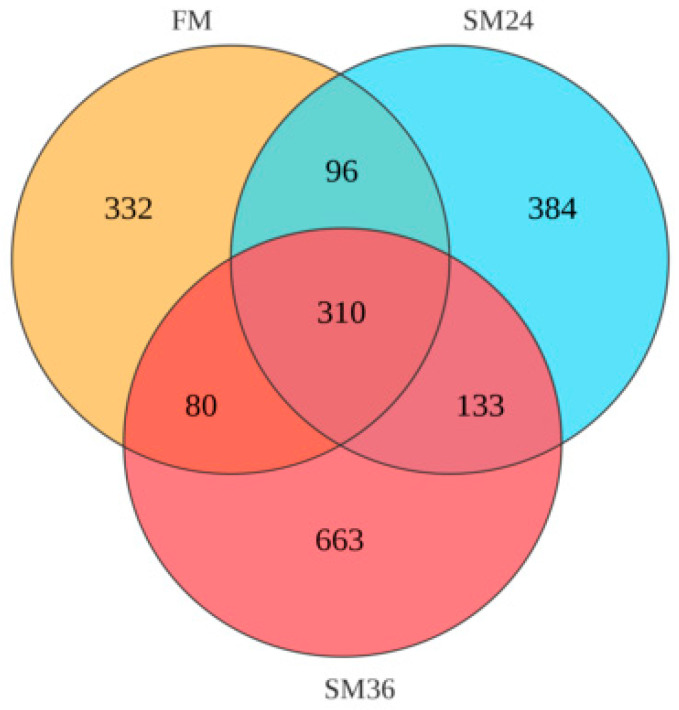
Venn diagram of intestinal microbiota.

**Figure 7:**
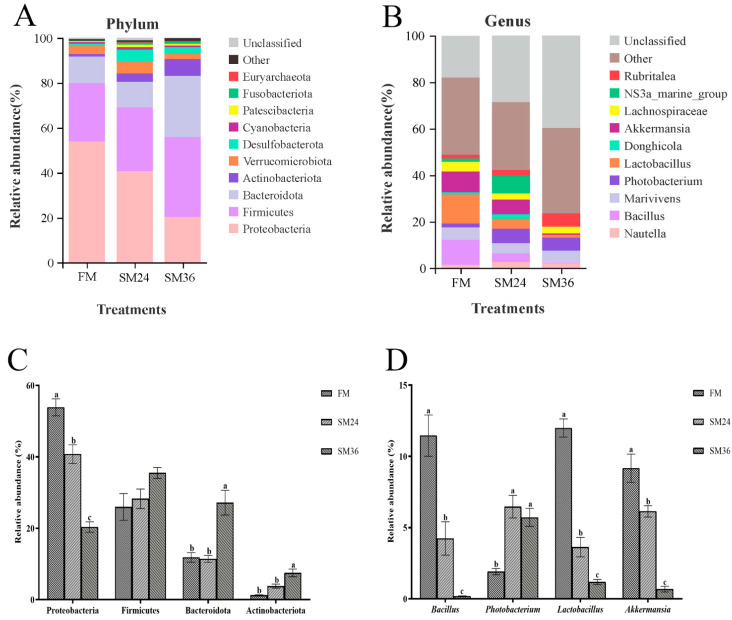
Intestinal microbiota profile of olive flounder fed with the experimental diets. (**A**) Top ten most abundant (based on relative abundance) bacterial phyla. (**B**) Top ten most abundant (based on relative abundance) bacterial genera. (**C**) Relative abundance of top four dominant bacterial phyla. (**D**) Relative abundance of top four dominant bacterial genera. Values are presented as the means ± standard error (*n* = 3). Different lowercases above the bars indicate significant differences (*p* < 0.05).

A Venn diagram showed that 310 OTUs were shared by the FM, SM24, and SM36 groups, and the number of unique OTUs in the FM, SM24, and SM36 groups was 332, 384, and 663, respectively (Figure 6). The composition of intestinal microbiota at the phylum and genus levels in olive flounder from the FM, SM24, and SM36 groups are presented in Figure 7. At the phylum level, Proteobacteria, Firmicutes, Bacteroidota and Actinobacteriota were identified as the predominant bacterial phyla in intestine of all the groups (Figure 7A). Compared with the FM group, the abundance of Proteobacteria decreased significantly in the SM24 and SM36 groups, whereas Bacteroidota and Actinobacteriota significantly increased in the SM36 group (*p* < 0.05). No significant difference in abundance of Firmicutes was observed among three groups (*p* > 0.05) (Figure 7C).

**Figure 8:**
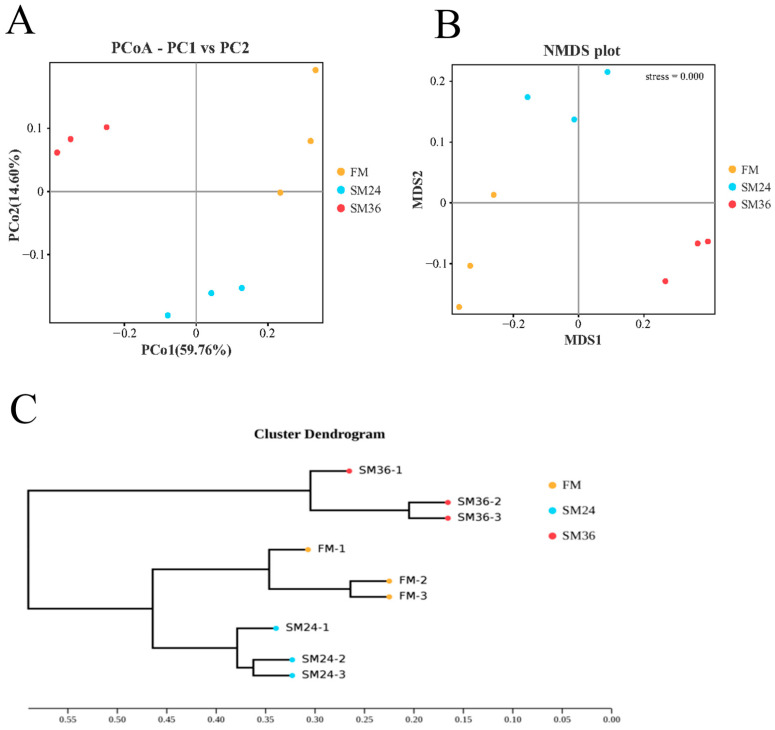
Comparison of intestinal microbiota of olive flounder fed with the experimental diets. (**A**) Principal coordinates analysis (PCoA) based on weighted UniFrac metrics. (**B**) Non-Metric Multi-Dimensional Scaling (NMDS) analysis based on weighted UniFrac metrics. (**C**) Unweighted Pair-group Method with Arithmetic Mean (UPGMA)-clustering trees based on weighted UniFrac distances.

At the genus level, *Nautella*, *Bacillus*, *Marivivens*, *Photobacterium*, *Lactobacillus*, *Donghicola*, *Akkermansia*, *Lachnospiraceae*, *NS3a-marine-group*, and *Rubritalea* composed the top ten dominant genera in intestine of olive flounder from the FM, SM24, and SM36 groups (Figure 7B). Compared with the FM group, the relative abundances of *Bacillus*, *Lactobacillus*, and *Akkermansia* in the SM24 and SM36 groups were significantly reduced (*p* < 0.05). The relative abundance of *Photobacterium* in the SM24 and SM36 groups was significantly increased (*p* < 0.05) (Figure 7D). The β-diversity analysis using PCoA (Figure 8A), NMDS (Figure 8B), and UPGMA (Figure 8C) indicated that the clusters and intestinal bacteria composition of the SM24 group were more similar to those of the FM group and distinctly separated from the SM36 group.

## 4. Discussion

Though substituting fishmeal in aquafeeds with plant-based protein sources holds promise as a means to advance sustainable aquaculture, the substitution level remains constrained due to the adverse impacts of plant proteins on carnivorous fish species. Our previous study demonstrated that substitution of fishmeal by SM exceeding 24% impaired growth performance, feed utilization, and muscle quality in olive flounder [24]. The present study evaluated the effects of replacing dietary fishmeal with SM on the serum biochemistry and intestinal health of olive flounder. The results indicated that when the replacement proportion of fishmeal by SM exceeded 12%, adverse effects on intestinal health were observed. Consistent with reports in other carnivorous fish species, such as turbot [31,32,33], Japanese seabass (*Lateolabrax japonicus*) [34], and gilthead sea bream (*Sparus aurata*) [35], high dietary SM appeared to disrupt multiple physiological processes, ultimately leading to enteropathy and systemic health issues. Serum biochemical indices serve as important indicators of fish health, which are closely related to intestinal nutrient absorption and health status, and they can change with nutritional state [36,37]. The TP content could reflect the efficiency of protein digestion and absorption metabolic status [38]. In our study, the serum TP content of olive flounder decreased significantly with increasing substitution level of fishmeal by SM, indicating that a high proportion replacement of dietary fishmeal with SM could inhibit the transport of metabolites and depress the rate of protein metabolism. Serum GLU level can reflect whether the nutrients in the diet can be better absorbed and utilized by the fish [39]. In this study, serum GLU level decreased in the SM36 and SM48 groups, potentially reflecting shift in energy utilization pathways due to SM-induced nutrient imbalances. Blood TG, TC, HDL-C, and LDL-C levels are important indicators reflecting the absorption and metabolism of lipids [40,41,42]. In this experiment, we found that when the proportion of SM replacing fishmeal exceeded 12%, the TG content in serum was significantly increased while HDL-C amount was significantly decreased, indicating that the fish may have some disorders in transport and metabolism of lipids. As an antimicrobial enzyme, LZM is an important non-specific immune indicator and plays a vital role in facilitating innate immunity [43,44]. Burrells et al. [45] reported that a high intake of SM suppressed non-specific immune response of rainbow trout (*Oncorhynchus mykiss*). In the present study, the serum LZM activity significantly decreased at substitution level exceeding 12%. Likewise, several studies have demonstrated that fish receiving diets with a high level of SM could lead to a reduction in LZM activity [33,34,46]. As a core organ responsible for digestion, absorption, and immune defense, abnormalities in the health status of intestine can affect systemic metabolism and organ functions through multiple pathways, which are further reflected in changes in serum biochemical parameters. Therefore, the variations in serum biochemical indices of olive flounder induced by replacing fishmeal with SM in this study may be related to intestinal dysfunction.

The intestine is a key organ responsible for digesting and absorbing nutrients from diets, and its morphological characteristics can directly reflect intestinal health [47]. Multiple studies have reported that excessive replacement of fishmeal with SM in diets of carnivorous fish induces intestinal inflammation and pathological changes [48,49]. Increased intestinal VH can expand the contact area with nutrients, thereby enhancing the efficiency of nutrient utilization [50]. In the present study, as the proportion of fishmeal replaced by SM increased, the intestinal VH decreased significantly. This suggested that a high level of dietary SM reduced intestinal surface area and impaired the capacity for nutrient digestion and absorption. Consistent with this, studies on orange-spotted grouper (*Epinephelus coioides*) [51] and Japanese seabass [7] also demonstrated that dietary SM significantly decreased intestinal VH. Similarly to our observations, replacement of fishmeal with SM significantly increased the width of lamina propria [52]. Goblet cells secrete mucins to form the intestinal mucin layer, which serves as the front line of innate host defense [53]. In the current study, replacing 48% of fishmeal with SM significantly reduced the density of intestinal goblet cells. Collectively, these findings indicated that high levels of dietary SM had adverse effects on the intestinal morphology of olive flounder.

The CAT and SOD are important components of the antioxidant defense system, which protects organisms against oxidative damage by scavenging reactive oxygen species. Their activities can reflect the antioxidant status of organisms [54]. Additionally, MDA is recognized as a final product of lipid peroxidation, and its level can also indicate the severity of endogenous oxidative damage [55]. In the present study, the T-AOC content and SOD activity in intestine decreased significantly as the substitution ratio of fishmeal by SM increased, while the MDA level in intestine of fish fed with the SM48 was significantly higher than that in other groups. Similarly, a high proportion replacement of dietary fishmeal with SM has been reported to induce an oxidative stress response in turbot, characterized by significantly reduced T-AOC amount and SOD activity as well as increased MDA content [56]. The declines in antioxidant enzyme activities and accumulation of MDA suggested that the intestinal antioxidant capacity of olive flounder was impaired when fed diets containing high levels of SM.

The SM-induced oxidative stress has been shown to arouse ERS, which can further induce inflammation when it persists [16,57]. The CHOP, PERK, and GRP78 are recognized as important marker proteins for ERS activation [58,59]. The IL-10 and TGF-β are well-known anti-inflammatory cytokines, whereas TNF-α and IL-6 are pro-inflammatory factors [60]. In the present study, the expression levels of *chop*, *perk*, and *grp78* in intestine increased markedly with increasing replacement levels of fishmeal by SM, indicating that high dietary SM could induce intestinal ERS in olive flounder. Meanwhile, high dietary SM significantly suppressed the gene expression of *il-10* and *tgf-β1* while elevating the expression of *tnf-α* and *il-6* in intestine. This aligns with previous studies showing that dietary SM can upregulate the expression of ERS-related genes and promote inflammation [13,61]. Inflammatory response is known to contribute to intestinal injury [62]. Zhu et al. [49] also demonstrated that a high percentage of dietary SM upregulated pro-inflammation cytokines and downregulated anti-inflammatory cytokines. Moreover, previous studies have shown that dietary SM can trigger intestinal inflammation by increasing the expression of pro-inflammatory cytokines [51,63,64].

In addition to inflammatory response, sustained ERS can also evoke apoptosis [57]. In general, apoptosis is an important mechanism for cell renewal, but excessive apoptosis can increase intestinal permeability and damage intestinal barrier [59]. Bax, a pro-apoptotic protein in the Bcl-2 family, promotes mitochondrial membrane permeability, thereby activating the caspase cascade and inducing apoptosis [65]. The caspase family ensures the orderly progression of apoptosis through complex activation mechanisms and cascade reactions [66]. In the current study, when SM replaced more than 12% of dietary fishmeal, the expression levels of *bax*, *casp3*, *casp6*, and *casp9* in intestine were significantly upregulated, indicating enhanced intestinal apoptosis in olive flounder. Consistent with this, a high proportion of dietary SM triggered apoptosis and impaired intestinal barrier in turbot and Nile tilapia (*Oreochromis niloticus*) [13,61].

The integrity of intestinal epithelial barrier function relies on the presence of healthy epithelial cells and properly functioning paracellular pathways [67]. Tight junctions are intercellular junctions that serve as cellular barriers in epithelial cells and regulate paracellular permeability [68,69]. The ZO, claudins, and occludin are important proteins composing tight junction [70], and reduced expression of these proteins impairs intestinal barrier function by increasing permeability [71,72]. In the present study, high dietary levels of SM significantly downregulated the mRNA expression of genes related to epithelial tight junction (*zo-1*, *zo-2*, *claudin-5*, and *occludin*), which might contribute to the increased intestinal permeability in olive flounder. Notably, claudin-15 mainly regulates Na^+^ transport and maintains intestinal ion balance through its cation-selective function in the paracellular pathway of intestinal epithelial cells [73]. However, dietary SM had no significant effect on intestinal *claudin-15* expression in the current study, which suggested that replacing fishmeal with SM may primarily impair the intestinal physical barrier function while exerting little impact on Na^+^ ion homeostasis in olive flounder.

The intestinal microbiota, regulated by various factors, with diet exerting a crucial role [74], plays a significant part in immunity, digestion, and metabolism, and is crucial for the normal physiology and health of the intestine [75,76,77]. Typical groups (FM, SM24, and SM36 groups) with significant differences in intestinal morphology and physiology were selected for intestinal microbiota analysis in the present study. Kim and Kim [78] reported that the dominant phyla in olive flounder were Proteobacteria, Firmicutes, Actinobacteria, and Bacteroideta. Consistent with this, our findings showed that Proteobacteria was the predominant phylum, followed by Firmicutes, Bacteroideta, and Actinobacteria. The relative abundances of Proteobacteria, Bacteroidota, and Actinobacteria changed significantly in the SM36 group. Similar results were also found in turbot [13], Atlantic salmon (*Salmo salar* L.) [79], and northern snakehead (*Channa argus Cantor*, 1842) [80]. Some marine Proteobacteria have been shown to produce bioactive compounds which own anti-cancer and antibiotic activity [81]. The remarkable decrease in Proteobacteria abundance in fish fed high dietary SM may be partially responsible for the impaired intestinal health status in this study. It has been reported that Bacteroidota possess the ability to break down complex carbohydrate [82], which helps to explain the significant enrichment of Bacteroidota in the SM36 group. A previous study demonstrated that high dietary SM exerted adverse effects on the gut-beneficial microorganisms (including *Bacillus* and *Lactobacillus*) [80]. In the current study, compared to the FM group, the SM24 and SM36 groups showed significantly reduced abundance of *Bacillus*, *Lactobacillus*, and *Akkermansia*, whereas boosted *Photobacterium* abundance in intestine. We speculated that high dietary SM might reduce the adhesiveness and biofilm activity of probiotics, thereby weakening their colonization in intestine of olive flounder. The *Lactobacillus* has been described to regulate intestinal epithelial barrier function via modulating the expression of adhesion junction proteins [83]. In addition, *Akkermansia* is a highly specialized bacterium capable of stimulating mucin expression and mucus secretion [84], whereas an increase in *Photobacterium* abundance may impair the intestinal immune mechanism of fish [85]. These results indicated that high-dose SM can cause intestinal microbiota dysbiosis, specifically characterized by the decrease in abundances of beneficial bacteria and increase in abundances of harmful bacteria.

In view of the abovementioned adverse effects triggered by replacement of dietary fishmeal with SM, exploring nutritional strategies, such as developing safe and environmentally friendly feed additives, to mitigate these detrimental impacts of low fishmeal diets on cultured fish is well imperative. There is increasing evidence suggesting that some feed additives or bioactive substances, such as *Lycium barbarum* polysaccharides [86], glutamine [13], daidzein [65], and resveratrol [87], can alleviate SM-induced enteropathy via relieving intestinal oxidative stress and inflammatory response, enhancing intestinal barrier function, and altering microbiota of fish. Our recent study revealed that dietary β-hydroxy-β-methylbutyrate (HMB) inclusion could improve growth and fillet texture of olive flounder fed diet with 36% of fishmeal substituted by SM [25], which prompted us to perform the next investigation about whether dietary HMB possesses a protective role on intestinal health of olive flounder fed a low fishmeal diet.

## 5. Conclusions

In conclusion, the feeding trial indicated that SM could replace up to 12% of dietary fishmeal without an adverse impact on intestinal health of olive flounder. Nevertheless, replacing 24% of dietary fishmeal with SM triggered fish intestinal damage. When the replacement level of fishmeal with SM reached 24% or higher, it significantly disrupted intestinal morphology, promoted the expression of genes related to ERS, inflammation, and apoptosis, while inhibiting the expression of tight junction-related genes. Meanwhile, there were significant differences in the abundances of intestinal microbiota at both the phylum and genus levels among the FM, SM24, and SM36 groups, but the clusters and intestinal bacterial composition of the SM24 group were more similar to those of the FM group. This study can provide a theoretical basis for the application of SM in diet of olive flounder.

## Figures and Tables

**Table 1:** Formulation and proximate composition of the experimental diets (% dry matter).

Ingredients	FM	SM12	SM24	SM36	SM48
Fishmeal	60.00	52.80	45.60	38.40	31.20
Soybean meal	0.00	10.38	20.77	31.15	41.54
Wheat meal	24.10	18.02	13.25	8.48	3.70
Wheat gluten	3.50	4.50	5.50	6.50	7.50
α-Starch	5.00	5.00	5.00	5.00	5.00
Fish oil	2.00	3.70	4.10	4.50	4.90
Soybean lecithin	1.50	1.50	1.50	1.50	1.50
Premix ^1^	1.00	1.00	1.00	1.00	1.00
Attractant ^2^	1.00	1.00	1.00	1.00	1.00
Methionine	0.00	0.08	0.16	0.24	0.32
Lysine	0.00	0.11	0.22	0.33	0.44
Others ^3^	1.90	1.90	1.90	1.90	1.90
Proximate composition					
Crude protein	50.24	50.35	50.28	50.13	50.18
Crude lipid	9.93	9.91	9.91	9.91	9.90
Ash	14.83	13.69	13.87	12.71	12.45

^1^ Warranty levels per kilogram of the product: retinyl acetate, 450,000 IU; cholecalciferol, 100,000 IU; ascorbic acid, 10,000 mg; inositol, 8000 mg; D, L-α-tocopherol acetate, 5000 mg; niacin, 3500 mg; calcium-D-pantothenate, 2000 mg; riboflavin, 700 mg; pyridoxine hydrochloride, 600 mg; menadione, 500 mg; thiamine nitrate, 500 mg; folic acid, 150 mg; D-biotin, 6 mg; cyanocobalamin, 2 mg; magnesium, 20,000 mg; zinc, 7500 mg; iron, 2000 mg; manganese, 2000 mg; copper, 1500 mg; iodine, 100 mg; cobalt, 80 mg; selenium, 10 mg. ^2^ Attractant: betaine:dimethyl-propiothetin:glycine:alanine:inosine 5′-monophosphate disodium salt = 4:2:2:1:1. ^3^ Others (%): chloride choline, 0.3; calcium propionate, 0.05; antioxidant, 0.05; sodium alginate, 0.50; Ca(H_2_PO_4_)_2_, 1.00.

**Table 3:** Serum biochemical parameters of olive flounder fed with the experimental diets.

Item	FM	SM12	SM24	SM36	SM48
TP (g/L)	67.57 ± 0.72 ^a^	61.42 ± 1.00 ^b^	55.52 ± 0.63 ^c^	51.53 ± 1.15 ^c^	49.27 ± 0.86 ^d^
ALB (g/L)	14.06 ± 0.47	14.58 ± 0.31	14.15 ± 0.32	14.12 ± 0.60	14.51 ± 0.45
GLU (mmol/L)	3.06 ± 0.07 ^c^	2.93 ± 0.12 ^c^	3.11 ± 0.18 ^c^	4.13 ± 0.12 ^b^	6.03 ± 0.10 ^a^
TG (mmol/L)	3.03 ± 0.11 ^c^	3.27 ± 0.16 ^c^	4.29 ± 0.11 ^b^	4.75 ± 0.12 ^b^	5.42 ± 0.17 ^a^
TC (mmol/L)	6.52 ± 0.03	6.42 ± 0.13	6.31 ± 0.13	6.29 ± 0.20	6.35 ± 0.16
HDL-C (mmol/L)	2.06 ± 0.05 ^a^	2.05 ± 0.09 ^a^	1.66 ± 0.04 ^b^	1.54 ± 0.07 ^b^	1.22 ± 0.03 ^c^
LDL-C (mmol/L)	3.67 ± 0.16 ^b^	3.49 ± 0.25 ^b^	3.63 ± 0.21 ^b^	3.50 ± 0.08 ^b^	4.76 ± 0.13 ^a^
LZM (U/mL)	207.06 ± 11.69 ^a^	188.30 ± 11.41 ^a^	146.75 ± 3.25 ^b^	130.10 ± 5.45 ^b^	127.50 ± 6.29 ^b^

Notes: Values are presented as the means ± standard error (*n* = 3). Values in the same row with different letters indicate significant differences (*p* < 0.05). TP, total protein; ALB, albumin; GLU, glucose; TG, total triglyceride; TC, total cholesterol; HDL-C, high-density lipoprotein cholesterol; LDL-C, low-density lipoprotein cholesterol; LZM, lysozyme.

**Table 4:** Intestinal antioxidant status of olive flounder fed with the experimental diets.

Item	FM	SM12	SM24	SM36	SM48
T-AOC (U/mg prot)	4.99 ± 0.09 ^a^	4.70 ± 0.09 ^ab^	4.59 ± 0.03 ^b^	3.37 ± 0.12 ^c^	2.52 ± 0.06 ^d^
CAT (U/mg prot)	4.39 ± 0.39	4.74 ± 0.33	4.78 ± 0.15	4.78 ± 0.26	4.88 ± 0.10
SOD (U/mg prot)	125.05 ± 2.79 ^a^	122.79 ± 2.30 ^ab^	121.91 ± 1.77 ^ab^	112.13 ± 2.16 ^bc^	103.22 ± 3.74 ^c^
MDA (nmol/mg prot)	1.74 ± 0.10 ^c^	1.69 ± 0.07 ^c^	2.09 ± 0.12 ^c^	2.76 ± 0.07 ^b^	3.61 ± 0.12 ^a^

Notes: Values are presented as the means ± standard error (*n* = 3). Values in the same row with different letters indicate significant differences (*p* < 0.05). T-AOC, total antioxidant capacity; CAT, catalase; SOD, superoxide dismutase; MDA, malondialdehyde.

## Data Availability

The original contributions presented in the study are included in the article, further inquiries can be directed to the corresponding authors.

## References

[1] Boyd C.E., D’Abramo L.R., Glencross B.D., Huyben D.C., Juarez L.M., Lockwood G.S., McNevin A.A., Tacon A.G., Teletchea F., Tomasso J.R. (2020). Achieving sustainable aquaculture: Historical and current perspectives and future needs and challenges. J. World Aquac. Soc..

[2] Wang J., Chen L., Xu J., Ma S., Liang X., Wei Z., Li D., Xue M. (2023). C1 gas protein: A potential protein substitute for advancing aquaculture sustainability. Rev. Aquac..

[3] Lam M.E. (2016). The ethics and sustainability of capture fisheries and aquaculture. J. Agric. Environ. Ethics.

[4] Grigorakis K. (2010). Ethical issues in aquaculture production. J. Agric. Environ. Ethics.

[5] Arriaga-Hernández D., Hernández C., Martínez-Montaño E., Ibarra-Castro L., Lizárraga-Velázquez E., Leyva-López N., Chávez-Sánchez M.C. (2021). Fish meal replacement by soybean products in aquaculture feeds for white snook, *Centropomus viridis*: Effect on growth, diet digestibility, and digestive capacity. Aquaculture.

[6] Liu X., Han B., Xu J., Zhu J., Hu J., Wan W., Miao S. (2020). Replacement of fishmeal with soybean meal affects the growth performance, digestive enzymes, intestinal microbiota and immunity of *Carassius auratus gibelio*♀ × *Cyprinus carpio*♂. Aquac. Rep..

[7] Zhang C., Rahimnejad S., Wang Y., Lu K., Song K., Wang L., Mai K. (2018). Substituting fish meal with soybean meal in diets for Japanese seabass (*Lateolabrax japonicus*): Effects on growth, digestive enzymes activity, gut histology, and expression of gut inflammatory and transporter genes. Aquaculture.

[8] Fuentes-Quesada J.P., Viana M.T., Rombenso A.N., Guerrero-Rentería Y., Nomura-Solís M., Gomez-Calle V., Lazo J.P., Mata-Sotres J.A. (2018). Enteritis induction by soybean meal in *Totoaba macdonaldi* diets: Effects on growth performance, digestive capacity, immune response and distal intestine integrity. Aquaculture.

[9] Wu N., Wang B., Cui Z., Zhang X., Cheng Y., Xu X., Li X., Wang Z., Chen D., Zhang Y. (2018). Integrative transcriptomic and microRNAomic profiling reveals immune mechanism for the resilience to soybean meal stress in fish gut and liver. Front. Physiol..

[10] Zhang J., Zhong L., Chi S., Chu W., Liu Y., Hu Y. (2020). Sodium butyrate supplementation in high-soybean meal diets for juvenile rice field eel (*Monopterus albus*): Effects on growth, immune response and intestinal health. Aquaculture.

[11] Volatiana J.A., Sagada G., Xu B., Zhang J., Ng W.K., Shao Q. (2020). Effects of butyrate glycerides supplementation in high soybean meal diet on growth performance, intestinal morphology and antioxidative status of juvenile black sea bream, *Acanthopagrus schlegelii*. Aquac. Nutr..

[12] Gu M., Jia Q., Zhang Z., Bai N., Xu X., Xu B. (2018). Soya-saponins induce intestinal inflammation and barrier dysfunction in juvenile turbot (*Scophthalmus maximus*). Fish Shellfish Immunol..

[13] Liu Y., Chen Z., Dai J., Yang P., Hu H., Ai Q., Zhang W., Zhang Y., Zhang Y., Mai K. (2018). The protective role of glutamine on enteropathy induced by high dose of soybean meal in turbot, *Scophthalmus maximus* L. *Aquaculture*
**2018**, *497*, 510–519. Aquaculture.

[14] Zhang Y., Duan X., Feng L., Jiang W., Wu P., Liu Y., Kuang S., Tang L., Zhou X. (2021). Soybean glycinin disrupted intestinal structural integrity related to aggravation of apoptosis and downregulated transcription of tight junction proteins in the intestine of juvenile grass carp (*Ctenopharyngodon idella*). Aquaculture.

[15] Cai M., Qiu X., Zhang H., Wang A., Xu W., Chen K., He Z., Hu Y. (2024). Effects of replacing fishmeal with soybean meal on the immune and antioxidant capacity, and intestinal metabolic functions of red swamp crayfish *Procambarus clarkii*. Fish Shellfish Immunol..

[16] Burgos-Morón E., Abad-Jiménez Z., Martinez de Maranon A., Iannantuoni F., Escribano-López I., López-Domènech S., Salom C., Jover A., Mora V., Roldan I. (2019). Relationship between oxidative stress, ER stress, and inflammation in type 2 diabetes: The battle continues. J. Clin. Med..

[17] Chaudhari N., Talwar P., Parimisetty A., Lefebvre d’Hellencourt C., Ravanan P. (2014). A molecular web: Endoplasmic reticulum stress, inflammation, and oxidative stress. Front. Cell Neurosci..

[18] Zhang B., Zhang Y., Cui M., Zhang M., Xu J., Zhang Z., Sui Z., Wang L., Zhang C., Li C. (2022). Comparison of the performance of raw and *Lactobacillus paracasei* fermented soybean meal in diets for turbot (*Scophthalmus maximus* L.): Growth, intestinal morphology, apoptosis, tight junction, and microbiota. Aquac. Rep..

[19] Cai M., Shao C., He Z., Chang R., Zhang H., Hu Y. (2025). Soybean meal-refined treatment mitigated high soybean meal diet-induced oxidative damage in the gut of crayfish via microbial metabolic function remodeling. Aquaculture.

[20] Pang A., Peng C., Xie R., Wang Z., Tan B., Wang T., Zhang W. (2023). Effects of fermented soybean meal substitution for fish meal on intestinal flora and intestinal health in pearl gentian grouper. Front. Physiol..

[21] Tremaroli V., Bäckhed F. (2012). Functional interactions between the gut microbiota and host metabolism. Nature.

[22] Zheng L., Zeng C., Zhu W., Zhang J., Wang L., Shao J., Zhao W. (2024). TLR2/TLR5 signaling and gut microbiota mediate soybean-meal-induced enteritis and declined growth and antioxidant capabilities in large yellow croaker (*Larimichthys crocea*). J. Mar. Sci. Eng..

[23] Egerton S., Wan A., Murphy K., Collins F., Ahern G., Sugrue I., Busca K., Egan F., Muller N., Whooley J. (2020). Replacing fishmeal with plant protein in Atlantic salmon (*Salmo salar*) diets by supplementation with fish protein hydrolysate. Sci. Rep..

[24] Shen N., Song Z., Xia C., Mu H., Chen X., Cheng H., Xu J., Sun Y., Wei C., Zhang L. (2024). Comparative evaluation of soybean meal vs. extruded soybean meal as a replacer for fishmeal in diets of olive flounder (*Paralichthys olivaceus*): Effects on growth performance and muscle quality. Aquaculture.

[25] Wei C., Su Z., Xia C., Li L., Song Z., Mu H., Cheng H., Xu J., Dong Z., Yan B. (2025). Effects of β-hydroxy-β-methylbutyrate inclusion on growth performance and fillet quality of olive flounder *Paralichthys olivaceus* fed low fishmeal based diets. Aquaculture.

[26] Livak K.J., Schmittgen T.D. (2001). Analysis of relative gene expression data using real-time quantitative PCR and the 2^−ΔΔCT^ method. Methods.

[27] Beck B.R., Song J.H., Park B.S., Kim D., Kwak J.H., Do H.K., Kim A.R., Kim W.J., Song S.K. (2016). Distinct immune tones are established by *Lactococcus lactis* BFE920 and *Lactobacillus plantarum* FGL0001 in the gut of olive flounder (*Paralichthys olivaceus*). Fish Shellfish Immunol..

[28] Callahan B.J., McMurdie P.J., Rosen M.J., Han A.W., Johnson A.J.A., Holmes S.P. (2016). DADA2: High-resolution sample inference from Illumina amplicon data. Nat. Methods.

[29] Quast C., Pruesse E., Yilmaz P., Gerken J., Schweer T., Yarza P., Peplies J., Glöckner F.O. (2012). The SILVA ribosomal RNA gene database project: Improved data processing and web-based tools. Nucleic Acids Res..

[30] Edgar R.C. (2004). MUSCLE: Multiple sequence alignment with high accuracy and high throughput. Nucleic Acids Res..

[31] Gu M., Bai N., Zhang Y., Krogdahl Å. (2016). Soybean meal induces enteritis in turbot *Scophthalmus maximus* at high supplementation levels. Aquaculture.

[32] Liu Y., Chen Z., Dai J., Yang P., Xu W., Ai Q., Zhang W., Zhang Y., Zhang Y., Mai K. (2019). Sodium butyrate supplementation in high-soybean meal diets for turbot (*Scophthalmus maximus* L.): Effects on inflammatory status, mucosal barriers and microbiota in the intestine. Fish Shellfish Immunol..

[33] Wang L., Zhou H., He R., Xu W., Mai K., He G. (2016). Effects of soybean meal fermentation by *Lactobacillus plantarum* P8 on growth, immune responses, and intestinal morphology in juvenile turbot (*Scophthalmus maximus* L.). Aquaculture.

[34] Rahimnejad S., Lu K., Wang L., Song K., Mai K., Davis D.A., Zhang C. (2019). Replacement of fish meal with *Bacillus pumillus* SE5 and *Pseudozyma aphidis* ZR1 fermented soybean meal in diets for Japanese seabass (*Lateolabrax japonicus*). Fish Shellfish Immunol..

[35] Kokou F., Sarropoulou E., Cotou E., Rigos G., Henry M., Alexis M., Kentouri M. (2015). Effects of fish meal replacement by a soybean protein on growth, histology, selected immune and oxidative status markers of gilthead sea bream, *Sparus aurata*. J. World Aquac. Soc..

[36] Dawood M.A., Koshio S., Ishikawa M., Yokoyama S., El Basuini M.F., Hossain M.S., Nhu T.H., Dossou S., Moss A.S. (2016). Effects of dietary supplementation of *Lactobacillus rhamnosus* or/and *Lactococcus lactis* on the growth, gut microbiota and immune responses of red sea bream, *Pagrus major*. Fish Shellfish Immunol..

[37] Van Doan H., Hoseinifar S.H., Dawood M.A., Chitmanat C., Tayyamath K. (2017). Effects of *Cordyceps militaris* spent mushroom substrate and *Lactobacillus plantarum* on mucosal, serum immunology and growth performance of Nile tilapia (*Oreochromis niloticus*). Fish Shellfish Immunol..

[38] Chen H., Luo D. (2023). Application of haematology parameters for health management in fish farms. Rev. Aquac..

[39] Ogunji J., Kloas W., Wirth M., Neumann N., Pietsch C. (2008). Effect of housefly maggot meal (magmeal) diets on the performance, concentration of plasma glucose, cortisol and blood characteristics of *Oreochromis niloticus* fingerlings. J. Anim. Physiol. Anim. Nutr..

[40] Chai Z., Yan Y., Zan S., Meng X., Zhang F. (2022). Probiotic-fermented blueberry pomace alleviates obesity and hyperlipidemia in high-fat diet C57BL/6J mice. Food Res. Int..

[41] Ding L., Zhang L., Wang J., Ma J., Meng X., Duan P., Sun L., Sun Y. (2010). Effect of dietary lipid level on the growth performance, feed utilization, body composition and blood chemistry of juvenile starry flounder (*Platichthys stellatus*). Aquac. Res..

[42] Su Y., Chen G., Chen L., Li J., Wang G., He J., Zhan T., Li Y., Yan M., Huang Y. (2019). Effects of antimicrobial peptides on serum biochemical parameters, antioxidant activity and non-specific immune responses in *Epinephelus coioides*. Fish Shellfish Immunol..

[43] Guo K., Ruan G., Fan W., Wang Q., Fang L., Luo J., Liu Y. (2020). Immune response to acute heat stress in the intestine of the red swamp crayfish, *Procambarus clarkii*. Fish Shellfish Immunol..

[44] Jia X., Qian P., Wu C., Xie Y., Yang W., Song R., Wu J., Ye J. (2022). Effects of dietary pantothenic acid on growth, antioxidant ability and innate immune response in juvenile black carp. Aquac. Rep..

[45] Burrells C., Williams P., Southgate P., Crampton V. (1999). Immunological, physiological and pathological responses of rainbow trout (*Oncorhynchus mykiss*) to increasing dietary concentrations of soybean proteins. Vet. Immunol. Immunopathol..

[46] Khosravi S., Rahimnejad S., Herault M., Fournier V., Lee C.R., Bui H.T.D., Jeong J.B., Lee K.J. (2015). Effects of protein hydrolysates supplementation in low fish meal diets on growth performance, innate immunity and disease resistance of red sea bream *Pagrus major*. Fish Shellfish Immunol..

[47] Rašković B., Stanković M., Marković Z., Poleksić V. (2011). Histological methods in the assessment of different feed effects on liver and intestine of fish. J. Agric. Sci..

[48] Krogdahl Å., Penn M., Thorsen J., Refstie S., Bakke A.M. (2010). Important antinutrients in plant feedstuffs for aquaculture: An update on recent findings regarding responses in salmonids. Aquac. Res..

[49] Zhu W., Yuan X., Luo H., Shao J., Chen X. (2021). High percentage of dietary soybean meal inhibited growth, impaired intestine healthy and induced inflammation by TLR-MAPK/NF-κB signaling pathway in large yellow croaker (*Larimichthys crocea*). Aquac. Rep..

[50] Krogdahl Å., Gajardo K., Kortner T.M., Penn M., Gu M., Berge G.M., Bakke A.M. (2015). Soya saponins induce enteritis in Atlantic salmon (*Salmo salar* L.). J. Agric. Food Chem..

[51] Wang Y., Wang L., Zhang C., Song K. (2017). Effects of substituting fishmeal with soybean meal on growth performance and intestinal morphology in orange-spotted grouper (*Epinephelus coioides*). Aquac. Rep..

[52] Li C., Tian Y., Ma Q., Zhang B. (2022). Dietary gamma-aminobutyric acid ameliorates growth impairment and intestinal dysfunction in turbot (*Scophthalmus maximus* L.) fed a high soybean meal diet. Food Funct..

[53] Cornick S., Tawiah A., Chadee K. (2015). Roles and regulation of the mucus barrier in the gut. Tissue Barriers.

[54] Radhakrishnan S., Bhavan P.S., Seenivasan C., Shanthi R., Muralisankar T. (2014). Replacement of fishmeal with *Spirulina platensis*, *Chlorella vulgaris* and *Azolla pinnata* on non-enzymatic and enzymatic antioxidant activities of *Macrobrachium rosenbergii*. J. Basic Appl. Zool..

[55] Ding Z., Zhang Y., Ye J., Du Z., Kong Y. (2015). An evaluation of replacing fish meal with fermented soybean meal in the diet of *Macrobrachium nipponense*: Growth, nonspecific immunity, and resistance to *Aeromonas hydrophila*. Fish Shellfish Immunol..

[56] Li C., Zhang B., Liu C., Zhou H., Wang X., Mai K., He G. (2020). Effects of dietary raw or *Enterococcus faecium* fermented soybean meal on growth, antioxidant status, intestinal microbiota, morphology, and inflammatory responses in turbot (*Scophthalmus maximus* L.). Fish Shellfish Immunol..

[57] Lemmer I.L., Willemsen N., Hilal N., Bartelt A. (2021). A guide to understanding endoplasmic reticulum stress in metabolic disorders. Mol. Metab..

[58] Zhang Y., Wei Z., Yang M., Liu D., Pan M., Wu C., Zhang W., Mai K. (2021). Dietary taurine modulates hepatic oxidative status, ER stress and inflammation in juvenile turbot (*Scophthalmus maximus* L.) fed high carbohydrate diets. Fish Shellfish Immunol..

[59] Zhao L., Liang J., Chen F., Tang X., Liao L., Liu Q., Luo J., Du Z., Li Z., Luo W. (2021). High carbohydrate diet induced endoplasmic reticulum stress and oxidative stress, promoted inflammation and apoptosis, impaired intestinal barrier of juvenile largemouth bass (*Micropterus salmoides*). Fish Shellfish Immunol..

[60] Neurath M.F. (2014). Cytokines in inflammatory bowel disease. Nat. Rev. Immunol..

[61] Wang T., Zhou N., He J., Hao Z., Zhou C., Du Y., Du Z., Su X., Zhang M. (2023). Xylanase improves the intestinal barrier function of Nile tilapia (*Oreochromis niloticus*) fed with soybean (*Glycine max*) meal. J. Anim. Sci. Biotechnol..

[62] Zhao X., Wang Y., Wang X., Ye J. (2021). Growth performance, plasma components, and intestinal barrier in grouper (*Epinephelus coioides*) are altered by dietary fish meal replacement with extruded soybean meal. Aquac. Rep..

[63] Hedrera M.I., Galdames J.A., Jimenez-Reyes M.F., Reyes A.E., Avendaño-Herrera R., Romero J., Feijóo C.G. (2013). Soybean meal induces intestinal inflammation in zebrafish larvae. PLoS ONE.

[64] Urán P., Gonçalves A., Taverne-Thiele J., Schrama J., Verreth J., Rombout J. (2008). Soybean meal induces intestinal inflammation in common carp (*Cyprinus carpio* L.). Fish Shellfish Immunol..

[65] Yu G., Liu Y., Ou W., Dai J., Ai Q., Zhang W., Mai K., Zhang Y. (2021). The protective role of daidzein in intestinal health of turbot (*Scophthalmus maximus* L.) fed soybean meal-based diets. Sci. Rep..

[66] Yu L., Yu H., Liang X., Li N., Wang X., Li F., Wu X., Zheng Y., Xue M. (2018). Dietary butylated hydroxytoluene improves lipid metabolism, antioxidant and anti-apoptotic response of largemouth bass (*Micropterus salmoides*). Fish Shellfish Immunol..

[67] Farhadi A., Banan A., Fields J., Keshavarzian A. (2003). Intestinal barrier: An interface between health and disease. J. Gastroenterol. Hepatol..

[68] Buckley A., Turner J.R. (2018). Cell biology of tight junction barrier regulation and mucosal disease. Cold Spring Harb. Perspect. Biol..

[69] Otani T., Furuse M. (2020). Tight junction structure and function revisited. Trends Cell Biol..

[70] Suzuki T. (2020). Regulation of the intestinal barrier by nutrients: The role of tight junctions. Anim. Sci. J..

[71] Guo G., Shi F., Zhu J., Shao Y., Gong W., Zhou G., Wu H., She J., Shi W. (2020). Piperine, a functional food alkaloid, exhibits inhibitory potential against TNBS-induced colitis via the inhibition of IκB-α/NF-κB and induces tight junction protein (claudin-1, occludin, and ZO-1) signaling pathway in experimental mice. Hum. Exp. Toxicol..

[72] He C., Deng J., Hu X., Zhou S., Wu J., Xiao D., Darko K.O., Huang Y., Tao T., Peng M. (2019). Vitamin A inhibits the action of LPS on the intestinal epithelial barrier function and tight junction proteins. Food Funct..

[73] Hempstock W., Nagata N., Ishizuka N., Hayashi H. (2023). The effect of claudin-15 deletion on cationic selectivity and transport in paracellular pathways of the cecum and large intestine. Sci. Rep..

[74] Brown K., DeCoffe D., Molcan E., Gibson D.L. (2012). Diet-induced dysbiosis of the intestinal microbiota and the effects on immunity and disease. Nutrients.

[75] Celi P., Cowieson A., Fru-Nji F., Steinert R., Kluenter A.M., Verlhac V. (2017). Gastrointestinal functionality in animal nutrition and health: New opportunities for sustainable animal production. Anim. Feed Sci. Technol..

[76] Liu S., Yu H., Li P., Wang C., Liu G., Zhang X., Zhang C., Qi M., Ji H. (2022). Dietary nano-selenium alleviated intestinal damage of juvenile grass carp (*Ctenopharyngodon idella*) induced by high-fat diet: Insight from intestinal morphology, tight junction, inflammation, anti-oxidization and intestinal microbiota. Anim. Nutr..

[77] Lu X., Tan K., Gong Q., Peng Y., Liang M., Xu P., Liang X., Liu W., Wu Y., Cai X. (2024). Positive effects of *Lithospermum erythrorhizon* extract added to high soybean meal diet on growth, intestinal antioxidant capacity, intestinal microbiota, and metabolism of pearl gentian grouper. Aquac. Rep..

[78] Kim D.H., Kim D.Y. (2013). Microbial diversity in the intestine of olive flounder (*Paralichthys olivaceus*). Aquaculture.

[79] Grammes F., Reveco F.E., Romarheim O.H., Landsverk T., Mydland L.T., Øverland M. (2013). *Candida utilis* and *Chlorella vulgaris* counteract intestinal inflammation in Atlantic salmon (*Salmo salar* L.). PLoS ONE.

[80] Miao S., Zhao C., Zhu J., Hu J., Dong X., Sun L. (2018). Dietary soybean meal affects intestinal homoeostasis by altering the microbiota, morphology and inflammatory cytokine gene expression in northern snakehead. Sci. Rep..

[81] Buijs Y., Bech P.K., Vazquez-Albacete D., Bentzon-Tilia M., Sonnenschein E.C., Gram L., Zhang S.D. (2019). Marine Proteobacteria as a source of natural products: Advances in molecular tools and strategies. Nat. Prod. Rep..

[82] Schwalm N.D., Groisman E.A. (2017). Navigating the gut buffet: Control of polysaccharide utilization in *Bacteroides* spp. *Trends Microbiol*. Trends Microbiol..

[83] Hummel S., Veltman K., Cichon C., Sonnenborn U., Schmidt M.A. (2012). Differential targeting of the E-Cadherin/β-Catenin complex by gram-positive probiotic lactobacilli improves epithelial barrier function. Appl. Environ. Microbiol..

[84] Fujio-Vejar S., Vasquez Y., Morales P., Magne F., Vera-Wolf P., Ugalde J.A., Navarrete P., Gotteland M. (2017). The gut microbiota of healthy Chilean subjects reveals a high abundance of the phylum Verrucomicrobia. Front. Microbiol..

[85] Mohamad N., Amal M.N.A., Yasin I.S.M., Saad M.Z., Nasruddin N.S., Al-saari N., Mino S., Sawabe T. (2019). Vibriosis in cultured marine fishes: A review. Aquaculture.

[86] Liu L., Zhao Y., Huang Z., Long Z., Qin H., Lin H., Zhou S., Kong L., Ma J., Lin Y. (2025). Dietary supplementation of *Lycium barbarum* polysaccharides alleviates soybean meal-induced enteritis in spotted sea bass *Lateolabrax maculatus*. Anim. Nutr..

[87] Tan C., Zhou H., Wang X., Mai K., He G. (2019). Resveratrol attenuates oxidative stress and inflammatory response in turbot fed with soybean meal based diet. Fish Shellfish Immunol..

